# Associations between ghrelin and ghrelin receptor polymorphisms and cancer in Caucasian populations: a meta-analysis

**DOI:** 10.1186/s12863-014-0118-3

**Published:** 2014-11-07

**Authors:** Noel A Pabalan, Inge Seim, Hamdi Jarjanazi, Lisa K Chopin

**Affiliations:** Center for Research and Development, Angeles University Foundation, Angeles City, 2009 Philippines; Graduate School, Cebu Doctors’ University, Mandaue City, 6014 Philippines; Research Office, Saint Louis University, Baguio City, 2600 Philippines; Ghrelin Research Group, TRI-Institute of Health & Biomedical Innovation, 37 Kent St., Woolloongabba, Brisbane, Queensland 4102 Australia; APCRC-Q, Queensland University of Technology, 37 Kent St., Woolloongabba, Brisbane, Queensland 4102 Australia; Environmental Monitoring and Reporting Branch, Ontario Ministry of the Environment, 125 Resources Road, Etobicoke, ON M9P 3 V6 2 Canada

**Keywords:** Ghrelin, *GHRL*, *GHSR*, Polymorphisms, Cancer

## Abstract

**Background:**

There is growing evidence that the ghrelin axis, including ghrelin (*GHRL*) and its receptor, the growth hormone secretagogue receptor (*GHSR*), play a role in cancer progression. Ghrelin gene and ghrelin receptor gene polymorphisms have been reported to have a range of effects in cancer, from increased risk, to protection from cancer, or having no association. In this study we aimed to clarify the role of ghrelin and ghrelin receptor polymorphisms in cancer by performing a meta-analysis of published case–control studies.

We conducted searches of the literature published up to January 2013 in MEDLINE using the PubMed search engine. Individual data on 8,430 cases and 14,008 controls from six case–control studies of an all Caucasian population were evaluated for three ghrelin gene (*GHRL*; rs696217, rs4684677, rs2075356) and one ghrelin receptor (*GHSR*; rs572169) polymorphism in breast cancer, esophageal cancer, colorectal cancer and non-Hodgkins lymphoma.

**Results:**

In the overall analysis, homozygous and recessive associations indicated that the minor alleles of rs696217 and rs2075356 *GHRL* polymorphisms conferred reduced cancer risk (odds ratio [OR] 0.61-0.78). The risk was unchanged for breast cancer patients when analysed separately (OR 0.73-0.83). In contrast, the rs4684677 *GHRL* and the rs572169 *GHSR* polymorphisms conferred increased breast cancer risk (OR 1.97-1.98, p = 0.08 and OR 1.42-1.43, p = 0.08, respectively). All dominant and co-dominant effects showed null effects (OR 0.96-1.05), except for the rs572169 co-dominant effect, with borderline increased risk (OR 1.08, p = 0.05).

**Conclusions:**

This study suggests that the rs696217 and rs2075356 ghrelin gene (*GHRL*) polymorphisms may protect carriers against breast cancer, and the rs4684677 *GHRL* and rs572169 *GHSR* polymorphisms may increase the risk among carriers. In addition, larger studies are required to confirm these findings.

## Background

It is appreciated that ghrelin and its receptor (members of the ghrelin axis) play a role in the development and progression of cancer [1]. Ghrelin, the endogenous ligand for the growth hormone secretagogue receptor (GHSR), has many functions, including a role in regulating growth hormone release [2] and a range of metabolic effects: regulating appetite, and influencing insulin and glucose homeostasis, energy balance and adipogenesis [3,4]. Given the metabolic effects of ghrelin, the ghrelin axis is a promising target for interventions for obesity and diabetes mellitus type two [5].

There is growing evidence that obesity and metabolic syndrome is associated with endocrine related cancers [6] and that the ghrelin axis may play a role in cancer progression [1]. A mechanistic link has been hypothesised between obesity, ghrelin and the development of colorectal [7] and prostate cancer [8]. A number of studies have linked single nucleotide polymorphisms (SNPs) in the ghrelin (*GHRL*) or ghrelin receptor (*GHSR*) genes with cancer risk [1]. Here, we perform a meta-analysis of case–control studies that have correlated ghrelin and *GHSR* gene polymorphisms with cancer risk to elucidate further the association between ghrelin axis gene polymorphisms and cancer.

## Methods

### Data sources

Using PubMed, a literature search was performed for all association studies (available until January 2013) investigating links between cancer and the ghrelin (*GHRL*) and ghrelin receptor (*GHSR*) genes. Previous studies reporting Caucasian genotypic data with case–control designs were chosen as eligible for this meta-analysis. In the first search, we used the terms, “*ghrelin*”, “*polymorphism”* and “*cancer*” which yielded 11 citations, five of which were excluded. From abstracts of the remaining six, one was excluded as it described an Asian population. Full texts of the remaining five studies were obtained, all of which complied with our inclusion criteria. In the second search, we entered the terms, “*GHRL*” and “*cancer*” in PubMed yielding 13 citations. A series of exclusions reduced the number to seven, full-texts of which were retrieved. We checked the reference lists of the full-text articles from both searches to minimize the possibility of missing relevant studies. Of the seven studies, six were either duplicated by the first search, or lacked genotype data, and therefore, only one further article was suitable for inclusion. Combining outcomes from the two searches gave a total of six articles which were included in our meta-analysis [9–14].

### Data extraction and power calculations

Two investigators independently extracted data and reached a consensus regarding all information. The following information was obtained from each publication: first author’s name, published year, country of origin, dominant ancestry of the study populations, state of controls, matching criteria, sample source, genotype data, number of cases and controls. We also calculated frequencies of the variant allele, deviations of controls from the Hardy-Weinberg equilibrium (HWE) and the statistical power of each study. Assuming an odds ratio (OR) of 1.5 at a genotypic risk level of α = 0.05 (two-sided), power was considered to be adequate at ≥80%.

### Meta-analysis

The strength of association between the ghrelin polymorphisms and cancer risk was measured by odds ratios (ORs) with 95% confidence intervals (CIs). Pooled estimates of the OR were obtained by calculating a weighted average of OR from each study [15]. For the following genetic models using variant (*var*) and wild-type (*wt*) genotypes we estimated: (i) **additive**: (*var-var*, var-var and *wt****-****wt*) genotypes compared with the *wt****-****wt*, (ii) **co-dominant**: frequency of variant alleles, assuming the risk could differ across all three genotypes, (iii) **recessive** (*var-var* vs. *wt-var* + *wt-wt*) and (iv) **dominant**: (*var-var* + *wt-var* vs. *wt-wt*).

To compare effects on the same baseline, we used raw data for genotype frequencies to calculate pooled ORs, which were obtained using either the fixed effects model [16], in the absence of heterogeneity, or random effects model in the presence of heterogeneity [17]. Heterogeneity between studies was estimated using the χ^2^-based Q test [18]. Given the low power of this test [19], significance threshold was set at P = 0.10. Heterogeneity between studies was estimated using the χ^2^-based Q test [18] and quantified with the I^2^ statistic which measures degree of inconsistency among studies [20]. Sensitivity analysis, which involved omitting one study at a time and recalculating the pooled OR, was also used to test for robustness of the summary effects. Data were analyzed using Review Manager 5.3 (Copenhagen: Nordic Cochrane Centre, Cochrane Collaboration) [21] and SigmaStat 2.3 (Systat Software, San Jose, CA). Significance was set at a P-value of ≤0.05 throughout, except in heterogeneity estimation. Publication bias was not investigated because of the low sensitivity of the qualitative and quantitative tests when the number of studies is lower than ten [22].

## Results

### Included studies

**Figure 1:**
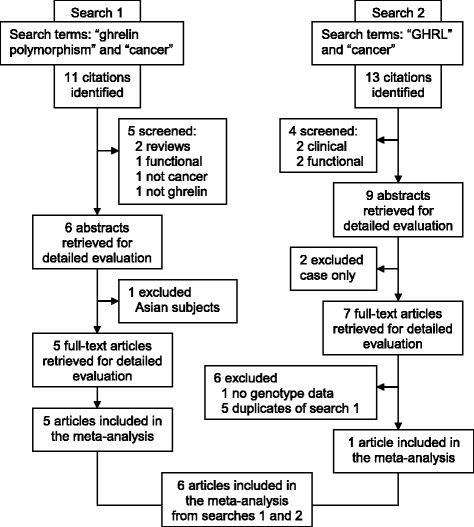
**Flowchart of literature search.**

**Figure 2:**
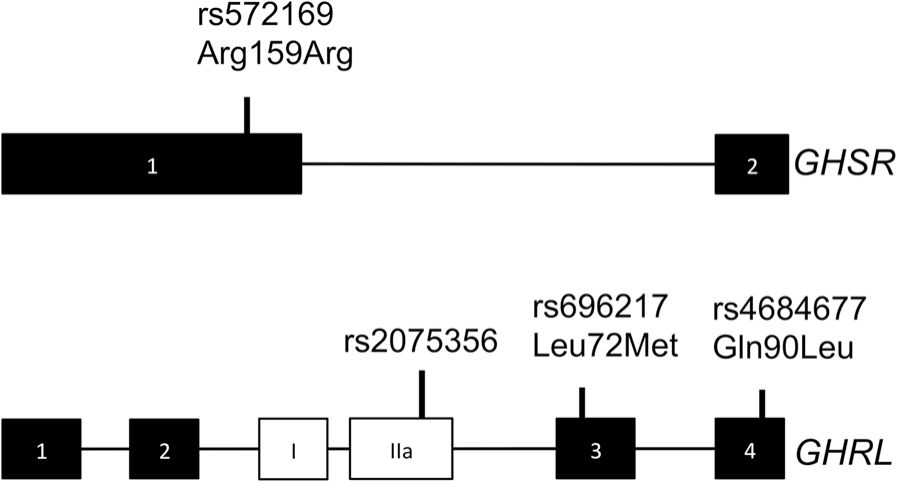
**Schematic diagram of the genes encoding ghrelin (** ***GHRL*** **) and the ghrelin receptor (** ***GHSR*** **).** Exons are shown as boxes, introns as lines. The canonical coding exons of GHSR1a (cognate ghrelin receptor; *GHSR*) and ghrelin (*GHRL*) are shown as black boxes. Exon I and II (white boxes) are unique to the *GHRL* splice variant in2c-ghrelin. The SNPs examined in this study are indicated.

A total of six genotyping studies [10–14,23] were included in the meta-analysis (Figure 1). The study features, which include nine ghrelin (*GHRL*) or ghrelin receptor (*GHSR*) single nucleotide polymorphisms (SNPs), the cancer type (breast, colorectal, esophageal and non-Hodgkin’s lymphoma) and study sample sizes*,* are outlined in Table 1. An overview of the ghrelin and ghrelin receptor SNPs examined are shown in Figure 2. Analyses of the pooled ORs revealed that five (rs495225, rs35684, rs27647, rs26802 and rs35683) of the nine SNPs investigated exhibited null effects in all genetic models (data not shown). The remaining three ghrelin SNPs (rs696217, rs4684677, rs2075356) and one *GHSR* SNP (rs572169) showed effects other than null, and were examined further. The features of these four SNPs (in six different studies), which included cancer type (breast, colorectal, esophageal, and non-Hodgkin’s lymphoma), ethnicity, number of cases and controls, calculated statistical power, minor allele frequency (MAF) and HWE are summarised in Table 2. The studies, that included rs696217 [10–14,23], rs4684677 [10–13], rs2075356 [11,12,23] and rs572169 [11,14,23] had statistical power of >83%, indicating that these studies were not underpowered (Table 2). Control frequencies in two component studies [10,14] deviated from the HWE in the rs4684677 and rs572169 polymorphisms (Table 2). Furthermore, three studies demonstrated borderline deviation from the HWE (p = 0.05-0.06) for the rs69621, rs2075356 and rs572169 polymorphisms [10,11,23].

**Table 1:** **The nine** ***GHRL*** **/** ***GHSR*** **polymorphisms examined in the meta-analysis, the number of studies performed in cancer samples and sample sizes**

			**N studies**							
	**Polymorphism**	**References**	**Cancer type**					**Number of**		
			**BC**	**CRC**	**EC**	**NHL**	**Total**	**Cases**	**Controls**	**Total**
1	***GHRL*** **696217**	[10–14,23]	3	1	1	1	6	3,601	6,101	9,702
2	***GHRL*** **4684677**	[10–12,23]	2	1	1	0	4	2,888	4,938	7,826
3	*GHRL* 27647	[11,12,23]	2	2	0	0	4	2,512	3,709	6,221
4	*GHRL* 26802	[11,12,23]	2	1	0	0	3	1,958	2,994	4,952
5	***GHRL*** **2075356**	[11,12,23]	2	1	0	0	3	1,941	2,972	4,913
6	*GHRL* 35684	[11,13,23]	1	1	0	1	3	2,144	3,480	5,624
7	*GHRL* 35683	[12,23]	1	2	0	0	3	1,864	1,949	3,813
8	*GHSR* 495225	[11,14,23]	2	1	0	0	3	2,350	3,370	5,720
9	***GHSR*** **572169**	[11,14,23]	2	1	0	0	3	2,378	3,414	5,792

Studies have examined ghrelin gene (*GHRL*) or ghrelin receptor gene (*GHSR)* polymorphisms in breast cancer (BC); colorectal cancer (CRC); esophageal cancer (EC); and Non-Hodgkin’s Lymphoma (NHL) [10–14,23].

SNPs in bold were examined in more detail in the current study.

**Table 2:** **Characteristics of the four** ***GHRL*** **/** ***GHSR*** **polymorphisms in six studies in breast cancer (BC), colorectal cancer (CRC), esophageal cancer (EC) and Non-Hodgkin’s Lymphoma (NHL)**

			**Number of**					
**First author (year)**	**Ethnicity**	**Cancer type**	**Cases**	**Controls**	**Total**	**Power (α = 0.05, OR = 1.5)**	**MAF**	**HWE**
rs696217 Leu72Met *GHRL* exon 3								
Dossus [11]	Europe	Breast	1,324	2,360	**3,684**	99.9	0.08	0.81
Feigelson [12]	USA	Breast	639	649	**1,288**	94.8	0.08	0.76
Wagner [14]	Europe	Breast	395	456	**851**	82.8	0.08	0.84
Campa [23]	Europe	CRC	678	600	**1,278**	94.6	0.07	0.05
Doecke [10]	Australia	EC	260	1,352	**1,612**	83.9	0.08	0.05
Skibola [13]	USA	NHL	305	684	**989**	82.7	0.08	0.59
rs4684677 Gln90Leu* GHRL *exon 4								
Dossus [11]	Europe	Breast	1,311	2,339	**3,650**	99.9	0.06	0.78
Feigelson [12]	USA	Breast	634	647	**1,281**	94.7	0.05	0.14
Campa [23]	Europe	CRC	683	600	**1,283**	94.6	0.07	0.83
Doecke [10]	Australia	EC	260	1,352	**1,612**	83.9	0.06	0.02
rs2075356 3056 T > C *GHRL* intron 3/in2c ghrelin 3’UTR								
Dossus [11]	Europe	Breast	634	1,734	**2,368**	99.1	0.09	0.93
Feigelson [12]	USA	Breast	640	652	**1,292**	94.9	0.10	0.72
Campa [23]	Europe	CRC	667	583	**1,250**	94.1	0.08	0.05
GHSR rs572169 Gly57Gly *GHSR *exon 1								
Dossus [11]	Europe	Breast	1,327	2,368	**3,695**	99.9	0.27	0.06
Wagner [14]	Europe	Breast	402	458	**860**	83.2	0.26	0.004
Campa [23]	Europe	CRC	649	588	**1,237**	93.9	0.28	0.77

Minor allele frequency (MAF); Hardy-Weinberg Equilibrium (HWE). UTR = untranslated region.

#### Quantitative effects

**Figure 3:**
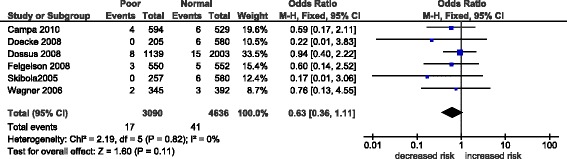
**Forest plot of homozygous pooled effect in the rs696217 polymorphism.** Black diamond denotes the pooled OR. Blue squares indicate the OR in each study, with square sizes directly proportional to the weight contribution (%) of the study. Horizontal lines represent 95% confidence intervals.

**Figure 4:**
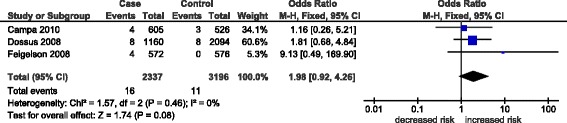
**Forest plot of homozygous pooled effect in the rs4684677 polymorphism.** Black diamond denotes the pooled OR. Blue squares indicate the OR in each study, with square sizes directly proportional to the weight contribution (%) of the study. Horizontal lines represent 95% confidence intervals. Note that case–control values for Doecke *et al*. [10] were non-estimable and therefore not included in the forest plot.

**Figure 5:**
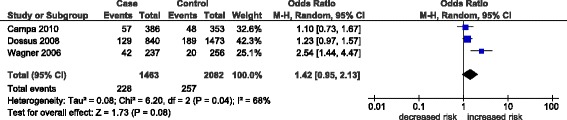
**Forest plot of homozygous pooled effect in the rs572169 polymorphism.** Black diamond denotes the pooled OR. Blue squares indicate the OR in each study, with square sizes directly proportional to the weight contribution (%) of the study. Horizontal lines represent 95% confidence intervals.

The overall effects (odd ratios) for the three *GHRL* polymorphisms (rs696217*,* rs4684677*,* rs2075356) and the rs572169 GHSR polymorphism, and the effects observed in breast cancer studies alone are shown in Table 3. Associations were observed mainly in the homozygous and recessive models and not in the dominant model, where the effects were null (OR 0.90-1.05, p = 0.19-0.92). Non-significant, decreased risks associated with the rs696217 (OR 0.61-0.63, p = 0.09-0.11) *GHRL* polymorphism in breast, colorectal, esophageal and colorectal cancer were not altered when analyses were confined to breast cancer studies alone (OR 0.82-0.83, p = 0.57-0.60) (Table 2). For rs696217*,* all of the study-specific ORs indicate reduced risk (Figure 3) and one study [11] in particular had a one-third weight contribution (33.5%) to the pooled effect (OR 0.63, p = 0.11). Similarly, a non-significant decrease in the risk of the *GHRL* polymorphism, rs2075356, in breast and colorectal cancer (OR 0.78, p = 0.43-0.45) was unaltered when confined to breast cancer studies (OR 0.78, p = 0.43-0.45) (Table 2). Increased risk in the *GHRL* SNP rs4684677 (OR 1.97-1.98, p = 0.08) associated with breast and esophageal cancers was exacerbated when confined to breast cancer (OR 2.38-2.40, p = 0.06). Figure 4 shows the contributions of study-specific ORs to the homozygous increased risk pooled effect of rs4684677 (OR 1.98, p = 0.08), mostly (60.6%) attributed to Dossus *et al.* [11]. On the other hand, the minimal weight contribution (5.3%) of the study by Feigelson *et al.* [12] is accompanied by wide confidence intervals (95% CI 0.49-169.90). The increased risk associated with the *GHSR* SNP rs572169 in breast and colorectal cancer (OR 1.42-1.43, p = 0.08) was also increased only when breast cancer was considered (OR 1.69-1.70, p = 0.14). While all of these effects of *GHRL* SNPs were obtained in zero heterogeneity (I^2^ = 0%), the effect of rs572169 was heterogeneous (I^2^ = 68-81%). Figure 5 shows heterogeneity (I^2^ = 68%) of the rs572169 increased risk pooled effect (OR 1.42, p = 0.08).

**Table 3:** **Summary odds ratios (OR) of associations between four ghrelin/GHSR gene polymorphisms with cancer using four genetic models (homozygous, dominant, recessive and co-dominant) in all studies analysed and in breast cancer studies alone**

		**Homozygous**			**Recessive**			**Dominant**			**Co-dominant**		
	**N**	**OR (95% CI) P value**	**P**_**het**_	**I** ^**2**^	**OR (95% CI) P value**	**P**_**het**_	**I** ^**2**^	**OR (95% CI) P value**	**P**_**het**_	**I** ^**2**^	**OR (95% CI) P value**	**P**_**het**_	**I** ^**2**^
rs696217	6	0.63 (0.36-1.11) 0.11	0.82	0	0.61 (0.35-1.08) 0.09	0.77	0	0.98 (0.87-1.11) 0.79	0.17	36	0.96 (0.86-1.07) 0.49	0.37	8
rs696217^a^	3	0.82 (0.41-1.62) 0.57	0.87	0	0.83 (0.42-1.65) 0.60	0.87	0	0.90 (0.78-1.05) 0.19	0.99	0	0.91 (0.79-1.04) 0.18	0.97	0
rs4684677	3	**1.98 (0.92-4.26) 0.08**	0.46	0	**1.97 (0.92-4.24) 0.08**	0.46	0	1.00 (0.86-1.16) 0.98*	0.28	22	1.02 (0.89-1.18) 0.75	0.28	23
rs4684677^a^	2	**2.40 (0.98-5.86) 0.06**	0.29	10	**2.38 (0.97-5.81) 0.06**	0.28	13	**1.08 (0.90-1.30) 0.39**	0.37	0	**1.11 (0.94-1.32) 0.23**	0.50	0
rs2075356	3	0.78 (0.41-1.47) 0.45	0.95	0	0.78 (0.41-1.46) 0.43	0.96	0	1.02(0.88-1.20) 0.76	0.58	0	1.02 (0.88-1.18) 0.79	0.56	0
rs2075356^a^	2	0.73 (0.33-1.62) 0.43	0.97	0	0.73 (0.33-1.62) 0.43	1.00	0	0.99 (0.83-1.19) 0.92	0.45	0	0.99 (0.84-1.18) 0.93	0.38	0
rs572169	3	**1.42 (0.95-2.13) 0.08**	0.04^**R**^	68	**1.43 (0.95-2.14) 0.08**	0.03^**R**^	70	**1.05 (0.94-1.16) 0.40**	0.82	0	**1.08 (1.00-1.18) 0.05**	0.34	7
rs572169^a^	2	**1.69 (0.84-3.41) 0.14**	0.02^**R**^	81	**1.70 (0.84-3.44) 0.14**	0.02^**R**^	82	**1.05 (0.93-1.18) 0.46**	0.53	0	**1.10 (1.00-1.21) 0.05**	0.17	46

^a^breast cancer only; N: number of studies; *N = 4; OR: odds ratio; CI: confidence interval; P_het_: P value for heterogeneity; the meta-analysis was conducted using the fixed-effects model unless otherwise stated; ^R^random-effects model (Bold indicates increased risk).

I^2^ values as measure of heterogeneity are considered low (<44%), moderate (45-74%) or high (>75%).

Increased risks were also observed in the co-dominant model of breast cancer studies for the *rs4684677 GHRL* SNP (OR 1.11, p = 0.23) and the *GHSR rs572169* SNP, both overall and in breast cancer (OR 1.08-1.10), with borderline significance (p = 0.05), all homogeneously obtained (I^2^ = 0-46%).

Omitting the studies that deviated from HWE (including those at borderline level) did not materially alter the original summary effects for all four polymorphisms (data not shown). Sensitivity analysis did not alter the effects of the rs4684677 and rs2075356 *GHRL* polymorphisms. Removal of the Dossus *et al*. [11] breast cancer study generated a significant protective effect of the *GHRL* SNP rs696217 in the recessive model (OR 0.46, 95% CI 0.21-0.97, p = 0.04). The omission of the breast cancer study by Feigelson *et al*. [12] erased heterogeneity for the *GHSR* SNP rs572169 (from I^2^ = 68% to 0%), but left the pooled OR materially unchanged.

## Discussion

With a combined sample size of 22,438 (8,430 cases and 14,008 controls), this meta-analysis provides evidence of overall homozygous and recessive associations, indicating a ~2-fold non-significant increase in cancer risk for the *GHRL* SNP rs4684677 and a ~1.4-fold non-significant increased risk for the *GHSR* SNP rs572169*.* This *GHSR* polymorphism showed a ~1.1-fold increased risk, with borderline significance in the co-dominant model. In contrast, the rs696217 and rs2075356 *GHRL* polymorphisms were both protective (22% and 38%), suggesting linkage disequilibrium (D’ = 0.90, r^2^ = 0.45) between the two SNPs [24]. The strength of these associations lie in the following: (i) they were obtained in total homogeneity underlying the statistical similarity of the component studies; and (ii) sensitivity analysis did not materially alter the effects underlying robustness of the pooled ORs. Interestingly, both the rs696217 (Leu72Met) and rs4684677 (Gln90Leu) *GHRL* SNPs have been linked with obesity [25,26]. There is growing recognition that obesity is a risk factor for a number of cancers, including breast, endometrial, colorectal, esophageal and prostate cancer [1].

The *GHRL* rs2075356 *(*3056 T > C) and *GHSR* rs572169 (Gly57Gly) SNPs were found to be associated with 20% increased risk of breast cancer in a European study with 1,324 cases and 2,360 controls [11]. These findings were similar in our meta-analysis, which included an additional 2,227 cases and 3,741 controls, for rs572169 with 1.7-fold increased breast cancer risk, however, rs2075356 was 27% protective in our meta-analysis. Ghrelin and ghrelin receptor are expressed in breast cancer tissue and ghrelin could play a role in breast cancer progression [1,27,28]. Interestingly, the *GHRL* SNP rs2075356 is present within the 3′ untranslated region of the recently discovered ghrelin transcript in2c-ghrelin, which is expressed in breast tumours, but not in normal breast tissue [8]. In2c-ghrelin is predicted to encode a novel 83 amino acid preprohormone that codes for the 28 amino acid ghrelin peptide (encoded by exon 1 and 2), but not obestatin (encoded by exon 3) [8]. Depending on the cell-type, obestatin has growth promoting or suppressing functions [29], however, the function of this peptide in breast cancer remains to be determined. The in2c transcript is insulin-regulated in prostate [8] and breast cancer cell lines (data not shown). Breast and prostate tumour cells are responsive to insulin [30–32], and elevated insulin (hyperinsulinaemia) is associated with breast and prostate cancer risk [33,34].

### Strengths and weaknesses

Each of the six component studies in our meta-analysis examined multiple polymorphisms of ghrelin and its receptors. The multiplicity of calculations involved necessitated statistical adjustment to avoid false-positive findings. All six studies were adjusted for multiple testing. Three used the conservative Bonferroni correction [10,12,23], one used the false positive report probability [11] and the fifth used the less conservative false discovery rate [13]. The sixth study did not test for multiplicity, but compared risk SNPs with a corresponding cohort study [14].

These correction procedures, as well as the aforementioned features of the cases and controls in the component studies, reflect the overall strength of this meta-analysis. Other strengths of this study include: (i) ethnic homogeneity of the subjects given our focus on Caucasians only, resulting in minimal admixture and control for potential effects of population stratification; (ii) high sample sizes translating to robust statistical power of the component studies; (iii) statistical homogeneity in the comparisons, so that data in the included studies were similar enough to be pooled. Moreover, (iv) findings in the breast cancer subgroup were obtained in zero heterogeneity; (v) controls were either healthy or cancer-free and were matched to cases; (vi) tissue sources were blood; and (vii) all component studies were population-based which minimizes effects of selection bias, such that findings may be extrapolated to the general population. Nonetheless, limitations that need to be acknowledged are: (i) lack of representation in the various cancers (except breast cancer) disallowed further subgroup comparisons; and (ii) deviation from HWE among controls of Skibola *et al.* [13] and Wagner *et al.* [14] in the rs4684677 and rs572169 SNPs, respectively.

## Conclusions

In summary, our results indicate that *GHRL* and *GHSR* SNPs may be involved in the pathophysiology of breast cancer. To our knowledge, this is the first meta-analysis to examine ghrelin polymorphisms and cancer risk. The demonstration of overall protective effects (of the rs696217 and rs2075356 *SNPs*) and increased susceptibility (for the rs4684677 and rs572169 *SNPs*) are derived from high-powered studies and are likely to increase the detection of low-penetrant effects. Further studies with larger and more well-defined sample populations are warranted to verify the role of *GHRL* and *GHSR* polymorphisms in cancer. This includes the analysis of additional metabolic, genetic and environmental contexts, which would be expected to influence the patient phenotype.
